# Supplementary material for: Metabolomics of in vivo inflammation identifies soluble sialic acid as a conserved myeloid-cell metabolite
Source: J Biol Chem. 2026 Sep 7;302(10):113519. doi: 10.1016/j.jbc.2026.113519 (PMC13659481; doi:10.1016/j.jbc.2026.113519)
Supplement: Supplementary file 1 [file mmc1.pdf]

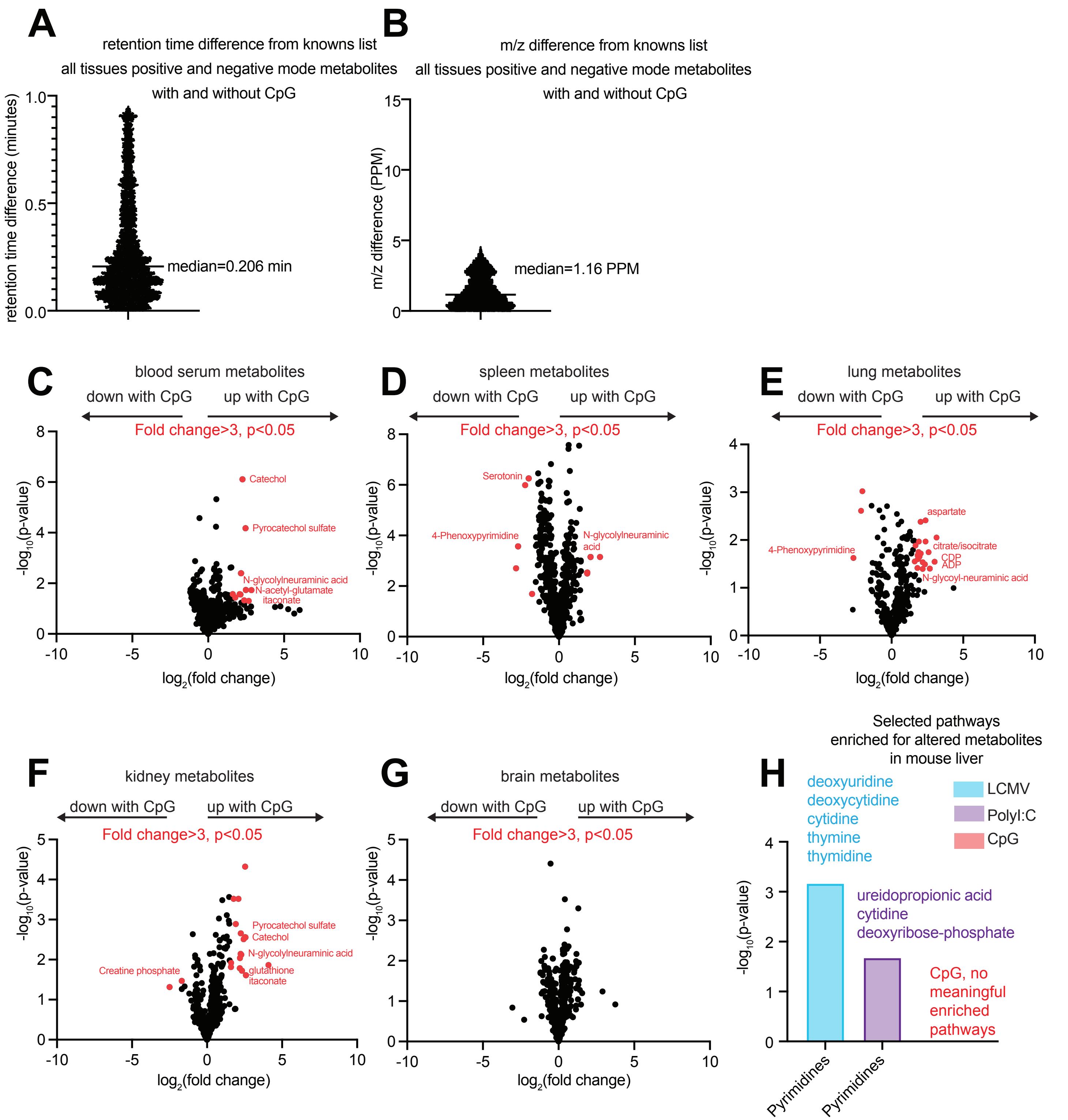

Figure S1, related to Figure 1: Metabolomic changes and pathway enrichments in inflamed mouse tissues. (A) Analysis of retention time difference between identified metabolites and historic standard compounds in 6 measured tissues of mice injected with CpG or control oligo, showing median difference is 0.206 minutes. (B) Analysis of m/z difference of each metabolite compared to its expected m/z in 6 measured tissues of mice injected with CpG or control oligo, showing median difference is 1.16 PPM (C-G): Metabolites changed between control mice and mice receiving intraperitoneal injection of 50 µg CpG DNA oligonucleotide (or control oligonucleotide) on days 0,2,4,6,8, with tissue collection on day 10, n=4 mice per group. (C) metabolites in blood serum, (D) spleen, (E) lung, (F) kidney, (G) brain; metabolites meeting the threshold of greater than 3-fold or less than 0.33-fold change, and $p<0.05$, are highlighted in red and selected metabolites are labeled, p-values were determined using a two-sided t test. (H) Pathways enriched for altered metabolites in mouse liver after 8 days of LCMV Clone 13 infection, 24 hours after polyI:C injection, or after 10 days of CpG injection.

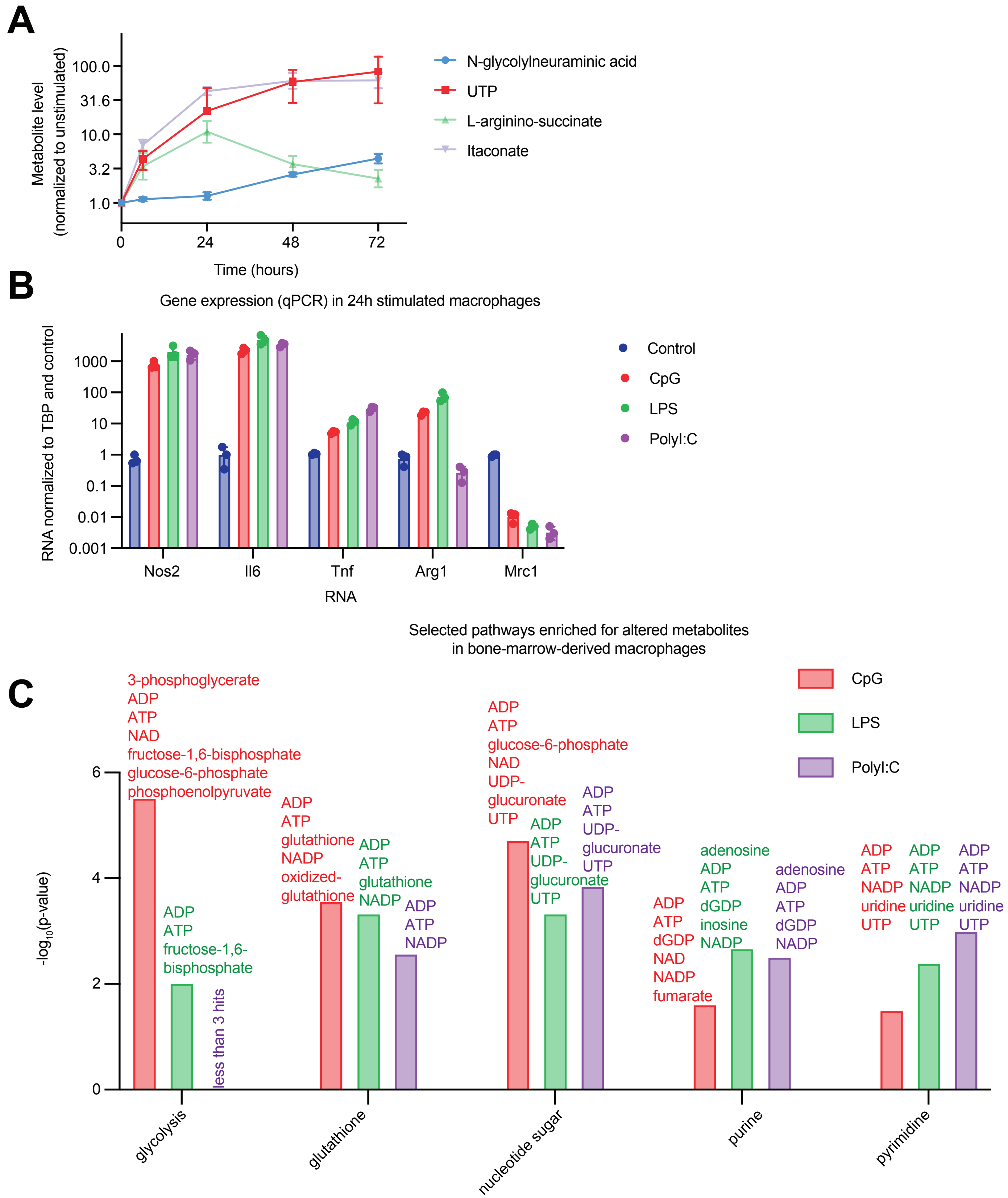

Figure S2, related to Figure 2: Metabolomic changes, gene expression changes, and pathway enrichments in stimulated bone-marrow-derived macrophages. A: Changes in selected metabolites at 0,4,24,48 or 72 hours after stimulation with 4ng/mL CpG oligonucleotide, showing that 24 hours is an early timepoint where large metabolite changes are detected. Error bars represent standard deviation. B: RT-PCR for selected genes in bone-marrow-derived macrophages 24 hours after stimulation with 4 ng/mL CpG DNA oligonucleotide, 4 ng/mL lipopolysaccharide (LPS), or 500ng/mL polyinosinic-polycytidylic acid (PolyI:C). Error bars represent standard deviation. C: Pathways enriched for altered metabolites in macrophages after 24 hours of stimulation with CpG, LPS or PolyI:C.

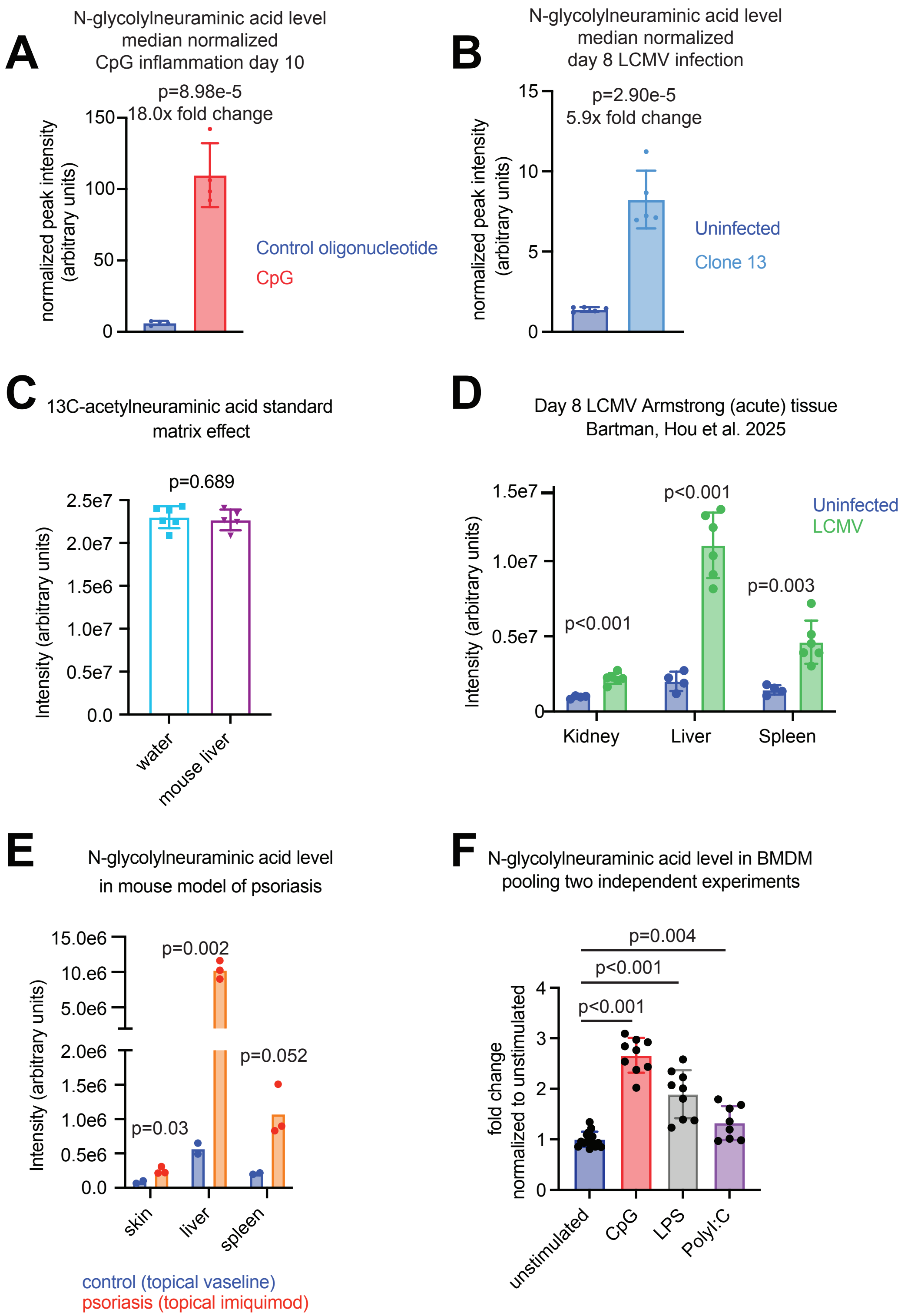

Figure S3, related to Figure 3: Inflammation increases N-glycolylneuraminic acid more in mouse tissues than in cultured macrophages. A: Median-normalized N-glycolylneuraminic acid level in livers of mice injected over 10 days with CpG or control oligonucleotide, n=4 mice per group. B: Median-normalized N-glycolylneuraminic acid level in livers of mice 8 days after infection with LCMV Clone 13 compared to uninfected mice, n=5 mice per group. C: Ionization of 13C-acetylneuraminic acid standard in water or mouse liver matrix, showing there is no significant matrix effect. D: N-glycolylneuraminic acid level from kidney, liver and lung of mice infected with acute Armstrong LCMV for 8 days, n=4 uninfected and n=6 infected mice, data from Bartman, Hou et al. 2025. C: N-glycolylneuraminic acid level in tissues of mice after 7 days topical application of 60mg imiquimod TLR7 agonist or vaseline control, n=2 control and n=3 imiquimod-treated mice. F: N-glycolylneuraminic acid level in bone-marrow-derived macrophages 24 hours after stimulation with 4 ng/mL CpG DNA oligonucleotide, 4 ng/mL lipopolysaccharide (LPS), or 500ng/mL polyinosinic-polycytidylic acid (PolyI:C), pooling two independent experiments, each normalized to unstimulated macrophages from same experiment. All p-values from two-sided t-tests; all error bars represent standard deviation.

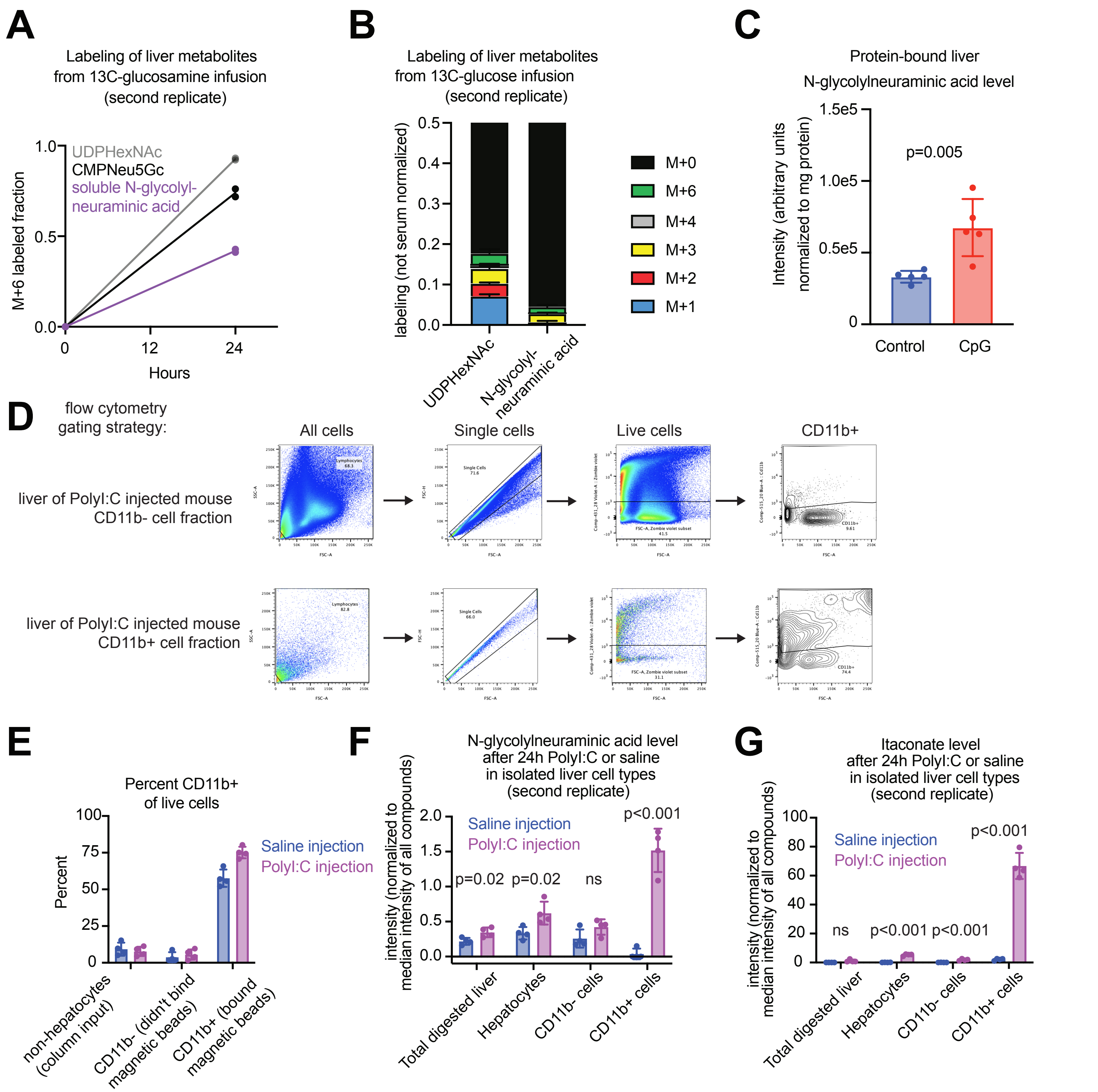

Figure S4, related to Figure 4: N-glycolylneuraminic acid is produced from sialylated proteins by CD11b+ myeloid immune cells in vivo. A : Mass+6 (m+6) carbon-13 enrichment of UDP-HexNAc, CMP-Neu5Gc, and soluble N-glycolylneuraminic acid in mouse liver after continuous infusion of m+6 13C-glucosamine for 0 or 24 hours, n=1 uninfused and n=2 24h-infused mice. B: Carbon-13 isotopologue distribution (m+0 through m+6) for UDP-HexNAc and soluble N-glycolylneuraminic acid after 24 hours of m+6 13C-glucose infusion, n=2 mice. C: Protein-bound N-glycolylneuraminic acid measured by protein isolation from mouse livers and acetic acid hydrolysis, in mice injected with control oligonucleotide or with CpG over 10 days, n=5 mice per group. D-E: Flow cytometry data to assess purity of liver CD11b+ myeloid cell isolation, related to data in Figure 4I; D: example gating strategy; E: CD11b+ percent of live cells from all samples. F: N-glycolylneuraminic acid level (normalized to metabolite median intensity) in digested liver, isolated hepatocytes, CD11b−non-hepatocytes, and CD11b+ cells from control and 24-hour PolyI:C-treated mice, n = 4 mice per group, second independent replicate of experiment in Figure 4I. G: Itaconate level (normalized to median metabolite intensity) in whole liver, isolated hepatocytes, CD11b− non-hepatocytes, and CD11b+ cells from control and 24-hour PolyI:C-treated mice, n = 4 mice per group. As expected, itaconate is enriched in CD11b+ myeloid cells. All p-values from two-sided t-tests; all error bars represent standard deviation.

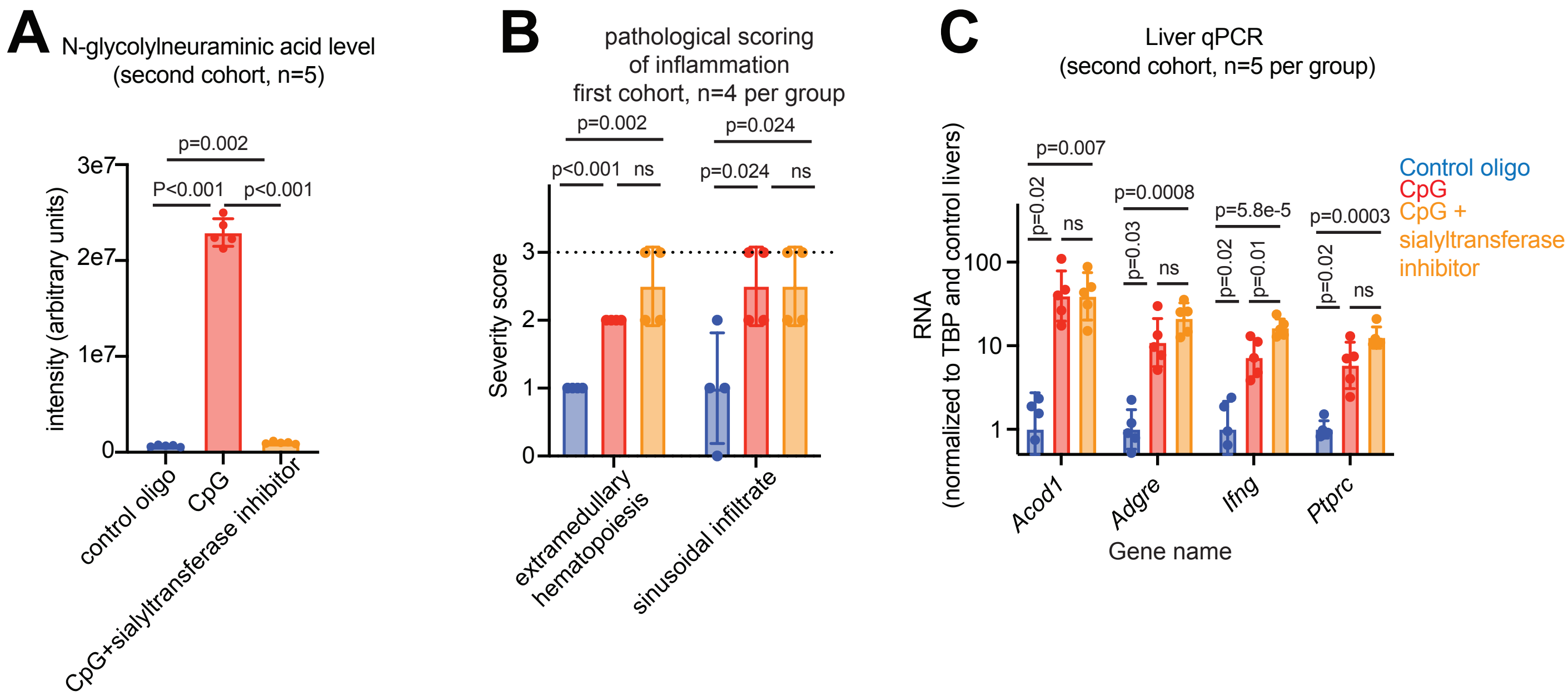

Figure S5, related to Figure 5: N-glycolylneuraminic acid does not affect pathology in CpG-induced cytokine storm. A : Soluble N-glycolylneuraminic acid level in livers of mice injected over 10 days with control oligonucleotide, CpG oligonucleotide, or CpG plus daily 30mg/kg P-3FAX-Neu5Ac sialyltransferase inhibitor injection, n=5 mice per group. B: Blinded pathologist scoring of liver inflammation in mice injected over 10 days with control oligonucleotide, CpG oligonucleotide, or CpG plus 30mg/kg P-3FAX-Neu5Ac sialyltransferase inhibitor, n=4 mice per group, second independent replicate of experiment in Figure 5C. C: Quantitative PCR of selected RNAs in livers of mice from Figure S5A, n=5 mice per group. in All p-values from two-sided t-tests; all error bars represent standard deviation.

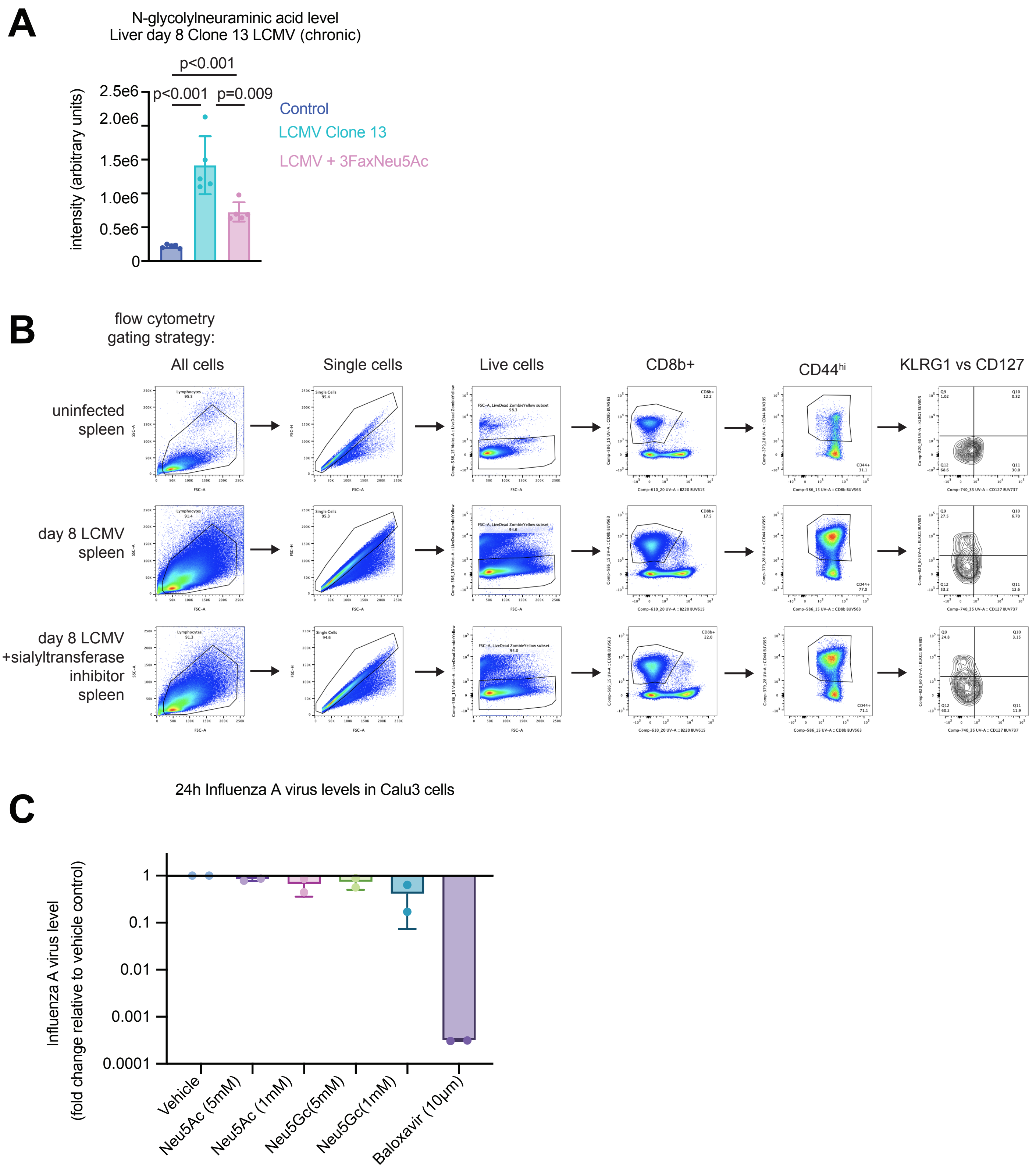

Figure S6, related to Figure 5: N-glycolylneuraminic acid does not affect viral infection.
A: Soluble N-glycolylneuraminic acid level in livers of uninfected mice, mice 8 days after LCMV Clone 13 infection, or LCMV Clone 13 plus daily 30mg/kg P-3FAX-Neu5Ac sialyltransferase inhibitor injection, n=5 mice per group, p-values from two-sided t-tests. B: Flow cytometry gating strategy to analyze CD8+ T cell phenotype, related to Figure 5G-H. C. Soluble N-glycolylneuraminic acid and N-acetylneuraminic acid do not reduce Influenza A virus infection in Calu-3 lung epithelial cells, showing n=2 independent experiments, each an average of 3 wells. Baloxavir (Influenza virus endonuclease inhibitor) is a positive control. All error bars represent standard deviation.

Supporting information tables:

Table S1: Fold changes and p-values of metabolomics datasets used in main and supplementary figures.
Table S2: Raw intensity values for each sample from metabolomics datasets used in main and supplementary figures.
